# Come Back Skinfolds, All Is Forgiven: A Narrative Review of the Efficacy of Common Body Composition Methods in Applied Sports Practice

**DOI:** 10.3390/nu13041075

**Published:** 2021-03-25

**Authors:** Andreas M. Kasper, Carl Langan-Evans, James F. Hudson, Thomas E. Brownlee, Liam D. Harper, Robert J. Naughton, James P. Morton, Graeme L. Close

**Affiliations:** 1 Research Institute for Sport and Exercise Sciences, Liverpool John Moores University, Liverpool L3 3AF, UK; a.kasper@2011.ljmu.ac.uk (A.M.K.); C.LanganEvans@ljmu.ac.uk (C.L.-E.); J.F.Hudson@2017.ljmu.ac.uk (J.F.H.); T.Brownlee@ljmu.ac.uk (T.E.B.); J.P.Morton@ljmu.ac.uk (J.P.M.); 2 School of Human and Health Sciences, University of Huddersfield, Huddersfield HD1 3DH, UK; L.Harper@hud.ac.uk (L.D.H.); R.Naughton@hud.ac.uk (R.J.N.)

**Keywords:** DXA, ultrasound, bioelectrical, impedance, scanning, plethysmography, densitometry, athlete, exercise, monitoring

## Abstract

Whilst the assessment of body composition is routine practice in sport, there remains considerable debate on the best tools available, with the chosen technique often based upon convenience rather than understanding the method and its limitations. The aim of this manuscript was threefold: (1) provide an overview of the common methodologies used within sport to measure body composition, specifically hydro-densitometry, air displacement plethysmography, bioelectrical impedance analysis and spectroscopy, ultra-sound, three-dimensional scanning, dual-energy X-ray absorptiometry (DXA) and skinfold thickness; (2) compare the efficacy of what are widely believed to be the most accurate (DXA) and practical (skinfold thickness) assessment tools and (3) provide a framework to help select the most appropriate assessment in applied sports practice including insights from the authors’ experiences working in elite sport. Traditionally, skinfold thickness has been the most popular method of body composition but the use of DXA has increased in recent years, with a wide held belief that it is the criterion standard. When bone mineral content needs to be assessed, and/or when it is necessary to take limb-specific estimations of fat and fat-free mass, then DXA appears to be the preferred method, although it is crucial to be aware of the logistical constraints required to produce reliable data, including controlling food intake, prior exercise and hydration status. However, given the need for simplicity and after considering the evidence across all assessment methods, skinfolds appear to be the least affected by day-to-day variability, leading to the conclusion ‘come back skinfolds, all is forgiven’.

## 1. Introduction

The assessment of body composition is routine practice in many sport organisations. Whilst total body mass (BM) assessments can be important in some situations (e.g., in sports where there is a given weight classification), the wider examination of body composition, specifically lean mass (LM) and fat mass (FM), is more informative for athletes and their coaches. This evaluation of FM, often reported as the percentage of the body that is fat (BF%), is highly relevant in many sports given that excess fat mass can be perceived as ‘dead weight’ when the body is resisting the forces of gravity in movements such as jumping and running. Despite the importance placed upon optimising and assessing body composition in elite sport, there is no universally accepted measurement method, with practitioners often selecting a technique that is suitable to their daily routines, as opposed to a thorough understanding of the methodologies and their limitations.

**Figure 1:**
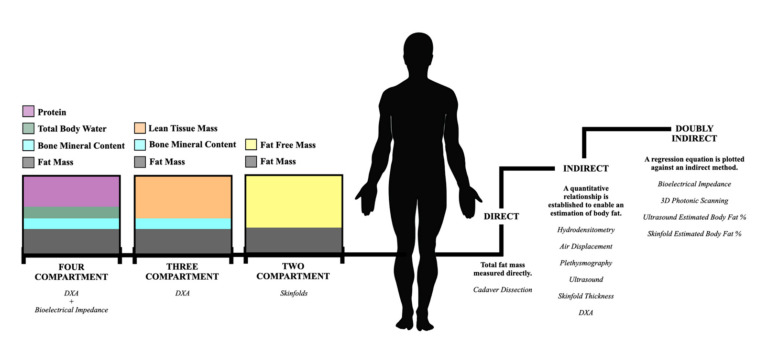
The 2-, 3- and 4-compartmental models of human body composition (left hand side), alongside the validation hierarchy (right hand side).

Throughout history, researchers attempted to study and accurately measure human anthropometry using a variety of techniques, ranging from early cadaver work to more recent imaging technologies such as dual-energy X-ray absorptiometry (DXA). In anthropometry, the body is often divided into compartments in a conceptual rather than anatomical separation. The simplest is the two-compartment model, which involves splitting the body into FM and fat-free mass (FFM), with the principle being that if one of these components is determined, the other can be estimated. The three-compartment model includes bone mineral content (BMC), FM and FFM (which is also inclusive of all other non-mineral tissues, i.e., organs), whilst the 4-compartment model also includes total body water (TBW) [1]. A visual representation of the compartment model is depicted in Figure 1.

Whilst various methods were developed to measure specific body tissues, cadaveric dissection is the only ‘direct’ method and therefore less invasive, more practical ‘indirect’ methods have been developed (Figure 1). Indirect methods include hydro-densitometry, which whilst accurate, has limited application in the elite sporting environment, as discussed below. Certain methods are deemed ‘doubly indirect’ and use predictive regression equations to quantify body composition based on an indirect technique, e.g., skinfold thickness measures with subsequent prediction equations. All of these methods may be deemed ‘suitable practice’ in specific situations and given there is no gold-standard body composition methodology in free-living individuals, it is crucial that athletes and coaches are fully aware of the various methodologies available and their limitations. Therefore, the aims of this current review are to (1) provide an overview of common methodologies used within sport to measure body composition; (2) compare the efficacy of what is widely believed to be the most valid (DXA) and most practical (skinfold thickness) measurement techniques within applied sports practice; and (3) provide a framework to help select the most appropriate body composition method in applied sports practice.

## 2. An Overview of Measurement Methodologies That Can Be Used in Applied Sport for the Assessment of Body Composition

Over the centuries, numerous techniques have been developed and utilised in an attempt to gain a greater understanding of the evaluation of body composition. However, the validity, accuracy, precision and reliability when employing some of these measures can be questionable, with methods often selected based on factors such as expense, safety, portability, invasiveness and requisite expertise for operation, rather than the most suitable for the required assessment. Whilst it is not possible to review every method of assessing body composition, this section will highlight those that have been used in the applied sport environment and critically evaluate their efficacy. Specifically, hydro-densitometry, air displacement plethysmography (ADP), bioelectrical impedance analysis and spectroscopy (BIA and BIS), ultrasound (US), three-dimensional (3D) scanning, DXA and skinfold thickness will be discussed. An overview of these techniques is provided in Table 1.

### 2.1. Hydro-Densitometry—Two Compartmental, Indirect

The indirect method of underwater weighing, termed hydro-densitometry, dates back to ~300 BC and uses assumed densities [2] and prediction formulae for determination of two-compartmental FFM and FM [2,3]. Historically, measures of body density established via underwater weighing have represented the ‘criterion’ for both accurate and reliable measurement of body composition and as a means for other methods to be validated against [4]. Employing the ‘Archimedes principle’ of measuring body volume, this technique is based upon the understanding that when an object is submerged underwater, the measurement of the ‘buoyant force’ is deemed to be equal to the weight of the water that it displaces [5]. When carried out by a qualified technician, the method can be accurate and reliable. However, limitations include (1) assumption of specific tissue densities, which may differ in elite athletic populations; (2) residual lung volume can be a source of error, given an individual must exhale all of their air whilst remaining static for a stable value; (3) some individuals may become claustrophobic whilst underwater or not be comfortable in water; (4) the equipment is now uncommon and costly, whilst needing to be maintained, sterilised and cleaned regularly; (5) the technique cannot measure distribution of FFM or FM; (6) air can be contained within an individual’s swimsuit, skin, head, and/or body hair or internally such as in their digestive tract, all of which can be included in false estimations [6]; (7) air temperature, barometric pressure, nitrogen analyser and force sensor calibrations all contain sources of error, which could contribute to the overall measurement error [7]; and (8) is time consuming and potentially uncomfortable. Despite the fact that hydro-densitometry has long been considered a criterion assessment of body composition, the emergence of accurate surrogate techniques are now seen as more suitable alternates in applied sport [8] and in laboratory-based sport science research [9,10,11].

### 2.2. Air Displacement Plethysmography—Two Compartmental, Indirect

Air displacement plethysmography (ADP) is an alternative to hydrostatic weighing and has more practicality in applied sport, where instead of water, air is utilised to measure body density. Some consider ADP to overcome several of the aforementioned limitations of hydro-densitometry [6]. Machines such as the BOD POD (COSMED, Rome, ITA), use Poisson’s Law to calculate air displacement and thus volumetric calculation. Isothermal air is then measured via inbuilt systems or generated via a prediction formula and combined to calculate a corrected body volume and body composition via numerous predictive equations [12]. Correlations of body density to assess validity when compared to hydro-densitometry have been found to be high [4,13,14]. Additionally, ADP has a high level of reliability (CV = 1.7 ± 1.1%) [4]. Despite these benefits, ADP is (1) insufficiently sensitive to detect in-competition changes in elite athletic body composition [15]; (2) sensitive to clothing, body hair, air movement, moisture, pressure and temperature changes [12,21,22,23]; and (3) expensive to purchase for those in the applied sports setting. When compared with hydro-densitometry, ADP overestimates BF% by ‘at least 1.28%’ [6]. When estimating adiposity in comparison to DXA, which will be covered later in this review, ADP diverges at the extremes of the BMI spectrum [24], which may be a cause for concern when used with certain athletic populations. Additionally, much like the use of hydro-densitometry, ADP is considered to be limited by its lack of ability to differentiate FM distribution, which may be of interest to practitioners. As such, this technique is not commonly used in applied sports practice and is primarily utilised in laboratory-based sport science research.

### 2.3. Bioelectrical Impedance Analysis and Spectroscopy—Multi-Compartmental, Doubly Indirect

Bioelectrical impedance methods are commonly used within general populations for assessment of body composition due to the speed of procedure, minimal expertise required to administer the test, portability and cost in comparison to other approaches. Indeed, it is not uncommon to see such units in gymnasiums and sports clubs. The methods of bioelectrical impedance are categorised by the number of frequencies used for analysis [25]. Techniques which are single frequency are commonly referred to as bioelectrical impedance analysis (BIA) devices (i.e., hand to hand), while multiple frequency methods are described as bioelectrical impedance spectroscopy (BIS) devices (i.e., hand and foot contact) [25]. BIS methods are considered superior to BIA methods as the calculation of body fluid volumes are based on Cole modelling and mixture theories as opposed to the simple regression equations used by BIA (for a more in-depth review see [25]). Despite this, the majority of previous research using bioelectrical impedance cites BIA methods. For both techniques, the currents are generated and measured using electrodes or metal contacts, which send a small voltage through the body to indirectly assess TBW volume. Based on the resistance to current flow observed within the body, FFM has more water and is less resistant than FM, which contains less water and is therefore more resistant. Bioelectrical impedance was previously validated for measuring TBW volume and may also distinguish between intracellular and extracellular fluid compartments [26,27]. Nevertheless, this method has several limitations including (1) outputs that can be affected by temperature and hydration status [28]; (2) sensitivity to conductive surface of electrodes and electrode placement [29]; (3) makes assumptions on the composition of the body in formula and calculations, irrespective of population group [30]; and (4) the limbs contribute a large proportion to whole-body impedance, despite the relatively low contribution to overall BM [31].

Within athletic populations, there are a limited amount of studies that have assessed the validation of bioelectrical impedance methods to measure FM and FFM, with conflicting findings when compared to DXA. Some investigations reported an underestimation of FM and overestimation of FFM [32,33,34,35,36], with others that reported overestimations of FM [37]. There is also large variability between devices [38], alongside large differences in the equations used to estimate body composition, making comparisons highly complex [25]. However, using bioelectrical impedance as a way of assessing change (provided methods are standardised) may be a useful tool [38] and is re-emerging in an applied sport context.

### 2.4. Ultrasound—Multi-Compartmental, Indirect (or Doubly Indirect If Prediction Equations Used)

A relatively new method in the measurement of body composition is ultrasound (US). This method measures uncompressed subcutaneous adipose tissue thickness through US imaging [39] by transmitting a high-frequency beam through the skin at a chosen surface anthropometry site(s) via a handheld transducer head. Once the beam meets an interface (such as subcutaneous fat or muscle tissues) an image is partially echoed back to the transducer, whereby specific types of tissues transmit differing acoustic impedance (i.e., FM content increases the time required for sound reflections off BMC to return to the probe [40]). An image can then be produced from the echo reflected back to the transducer, allowing the integrated software to produce an estimation of the thickness of the tissue (for a more in-depth review see [39]). The use of US can be an accurate measure of subcutaneous adipose tissue; however, it can be altered by (1) the chosen US frequency; (2) pressure and orientation of the probe causing measurement error; (3) the technician’s ability to choose the representative sites for measurements. Furthermore, US can be expensive and impractical within the applied setting, even with improved portability. One reported advantage of US is that intra-abdominal fat can be assessed more reliably than anthropometric measures [41], although, whilst this is useful to assess metabolic risk factors for cardiovascular disease, it is not routinely required in applied sport.

With regards to the application of US in applied sport, it is considered a reliable [42] and accurate measure [39] of subcutaneous adipose tissue thickness, but is limited by the plasticity of fat tissue and uneven tissue borders. More recently, Gomes et al. [43] reported US provides similar results for FM in comparison to skinfold and DXA across a range of athletes. However, the aforementioned study used a high-resolution B-mode US unit, which can be time-consuming (>20 min) and requires costly medical devices (>£11,000) along with expensive analysis software (>£1000) [44], that limits the application of such a method in an applied setting. Within an applied context, one of the more widely used methods is A-mode US devices which are commercially available at a considerably lower cost (such as the BodyMetrix^®^ (Intelmetrix, Brentwood, CA, USA) device (<£2000)). However, research on the validity of such devices is at best equivocal, with poor agreeability reported in comparison with DXA in both non-athletic [45,46,47] and athletic young adults [48], and when compared to BOD POD in NCAA Division I athletes [44]. Moreover, Peréz-Chirinos Buxadé and colleagues [49] reported that the A-mode US devices produced significantly lower skinfold thickness scores in comparison with skinfold caliper measures performed by The International Society for the Advancement of Kinanthropometry (ISAK) qualified technicians. In terms of the reliability of A-mode devices, mixed results have been reported, from excellent [44,50], to acceptable [51] and also poor [49]. However, it is important to note there are a range of devices available and results may be specific to the device assessed. It should also be noted that the US method involves converting uncompressed subcutaneous adipose tissue thickness into a percentage body fat using regression equations, which adds another layer of inaccuracy that is discussed in more detail in Section 3.2. One advantage of the US device is that it has better inter-rater reliability than skinfolds in novice (non ISAK trained) practitioners [52]. Whilst the use of portable US may prove an exciting avenue for assessment of body composition in the future, this requires further development and research to assess the accuracy and reliability of available devices; however, it could be an effective tool to produce repeatable data when there is no access to suitably trained skinfold practitioners (see Section 2.7).

### 2.5. 3D Photonic Scanning—One Compatmental, Doubly Indirect

The use of 3D scanners, a form of digital anthropometry, originates from the assessment of human body shape for garment manufacture [53]. Three-dimensional scanners are now used for a variety of purposes, including assessment of body composition. Briefly, data with a 3D scanner involves the use of visible and infrared light to create an avatar of the human body, with the subject required to stand still in a particular posture whilst wearing minimal clothing. The reflection of the light off the body allows for a series of points to be captured with triangulation [54]. These points are connected to create a 3D mesh, with the use of landmarks to calculate circumferences, volumes, lengths and surface areas (for a more in-depth explanation of data acquisition and processing, see [55]). In comparison to DXA and computerised tomography (CT) scans, 3D scanners do not require ionising radiation or principal component analysis, the outcome can be used to create a pseudo-DXA scan [55]. The use of 3D scanners is time efficient (a scan takes approx. 10 s), which provides advantages over other time-consuming methods. Theoretically, this method could be employed on a regular basis within athletic populations for frequent assessments of body composition, providing visualisations for retrospective comparisons. Conversely, the cost of using 3D scanners and the operative expertise required may make it a prohibitive method in most applied settings.

The first published data on the use of 3D scanners to assess the body composition of athletes were by Schranz et al. [56], who compared elite Australian rowers to age-matched non-athletic controls. They observed elite rowers had greater segmental volumes and cross-sectional areas, variables which cannot be measured with a one compartment method. Therefore, the authors suggested for talent identification purposes, 3D scanning may be implemented in the testing of potential athletes, with the same research group subsequently observing that 3D methods are better than 1D methods for predicting junior rowing performance [57]. Evidence exists for the accuracy and reliability of 3D scanning when estimating body composition [56,58,59,60], although there are differences between commercially available scanners, which is partly due to the differences in the algorithms used [60]. Indeed, the development and refinement of the most valid algorithm and post-processing technique are still required [61]. Nonetheless, not all data support the validity of 3D scanning [62,63]. Cabre et al. [63] observed high typical errors and significant under and over predicting of BF%, FM and FFM using 3D scans compared to a four component model (combined DXA, ADP and BIS) in a non-athletic population. However, the authors observed no significant differences between 3D and DXA measures. Conversely, Tinsley et al. [60] observed high limits of agreement (LoA) for BF% (7–9.5%) and FM/FFM (5.3–7.2 kg) when compared to a four component model. As these LoA are in excess of expected changes from typical dietary and exercise interventions, longitudinal data that compare 3D scanning to other methods of body composition assessment during interventions aimed to alter body composition are required. In summary, given the expense and lack of athlete-specific validation data, this technique is uncommon in applied sport and is mainly suitable for laboratory-based research.

### 2.6. Dual-Energy X-ray Absorptiometry—Three Compartmental, Indirect

Whilst DXA was first developed for the measurement of bone mineral density (BMD), it has been extensively utilised in athletic populations for the assessment of body composition [11,64,65,66]. Indeed, DXA is now considered by many in the field as the ‘criterion standard’ of body composition assessment, despite being used in clinical settings for the diagnosis of bone related disorders such as osteoporosis. DXA operates by passing both high and low energy x-ray photons in either a pencil or fan-based beam, through differing body regions. The energy of these beams is attenuated by the density and volume of differing tissues, with soft tissues such as FM and LM allowing greater passage of photons when compared with denser tissues such as bone. The system software then produces an image in a rectilinear fashion and measurements of cross-sectional areas are completed with quantification of FM, LM and BMC in a two-dimensional image, calculated from the coefficient of two differing peaks in order to generate an R-value. For an further in depth description of the technical aspects of DXA measurement in differing systems, readers are directed to a recent review by Bazzocchi and colleagues [67]. Although DXA can be a reliable measure of body composition [68,69], utilisation of this method is not without its limitations, inclusive of legal and ethical constraints and technical considerations that will be discussed in more detail in subsequent sections. The major strength of DXA is the ability to measure BMC, which is growing in importance in due to the increasing awareness of low energy availability and the consequences of this on bone mineral content [70]. Furthermore, DXA provides limb-specific estimations of FM and FFM which can be useful when tracking injured athletes and the magnitude of fat loss in weight-making athletes [9,10].

### 2.7. Skinfold Thickness—Two Compartmental, Indirect (or Doubly Indirect If Prediction Equations Used)

Skinfold thickness assessment involves the use of a caliper to measure a double fold of gripped skin, over a range of differing sites to establish an overall measurement of subcutaneous adiposity [71]. This method is an inexpensive technique, requiring minimal equipment (calibrated calipers and anthropometric tape measure), allowing assessment to be conducted in a number of different field-based settings making it a popular method for estimating FM [71,72]. Detailed methodology of specific protocols are outlined in a number of texts [73,74]. The number of anatomical sites measured and equations used to predict both body density and FM using this technique varies significantly, which can create discrepancies in the data collected. As a consequence, ISAK was founded in 1986 to provide training courses and accreditation worldwide, setting professional standards for using skinfold thickness to assess body composition, with the eight site method now considered by many as best practice in applied sport settings. Although traditionally the most popular and suitable method for field testing, this doubly indirect method was previously deemed unsuitable for the assessment of FFM and estimation of BF% [30]. The limitations and practical application of this technique will be covered in more detail in Section 3.

## 3. Practical Considerations When Using DXA and Skinfolds as Measures of Body Composition in Applied Sport Practice

Although traditionally skinfold thickness measurement has been the most popular method of body composition assessment in applied settings, DXA assessment has become increasingly more common in recent years [68], most likely due to a greater availability of machines and a belief that this is now the criterion standard. Despite these methods being used extensively in applied practice, both techniques produce outcomes based on a number of assumptions and require a high degree of standardisation for both accurate and reliable assessment, which is often ignored or not considered in applied sport settings. The following sections will serve to draw attention to these considerations, in the context of an applied sport setting, whilst providing a framework for best practice should these methods be considered and utilised.

### 3.1. DXA

The following practical considerations when using DXA to assess body composition are important to generate valid and reproducible data: (1) machine and software types, (2) legal and ethical considerations and (3) technical standardisation.

#### 3.1.1. Machine and Software Types

As highlighted earlier, DXA was originally developed to measure BMD within the general population and therefore quantifies FM and LM as a secondary measure. DXA scanning assesses the composition of photographic pixels, directly distinguishing bone (usually 40–45% of pixels) from other soft tissues (FM and LM). The remaining pixels are used to calculate the remaining soft tissue using a FM:LM ratio [75]. Where no bone is present, the ratio of the attenuation of the two beams is linearly proportional to fat within the soft tissue, with this relationship used to estimate FM and LM, respectively [76]. There are also variations between manufacturers, such as energy levels emitted, pixel size, beam path, software algorithms and scanning frequencies [67].

A further consideration often not considered are both inter-machine and inter-manufacturer variability, causing issues with athletes who are required to travel and may be scanned at different locations. Additionally, even when the same model of DXA machine is used, results may vary significantly between hardware and software version. For example, Table 2 shows data collected on elite female soccer players assessed on units produced by the same DXA manufacturer, yet with different models. These measurements were made within weeks of each other and despite players reporting with the same total BM, there was an 18% increase in FM and a 4% decrease in LM. Furthermore, the algorithm used within differing software packages can be modified with numerous iterations available for scan analysis, all of which may produce conflicting values. Within the published literature it is not common for authors to report which algorithm they have used for analysis, rather, only the make and model of machinery used. Furthermore, the software on DXA scanning systems often only allow allocation of athletes to a single racial classification, thereby not allowing further options for individuals who may be of multi-ethnic backgrounds. Such issues do not detract from the use of DXA; however, they must be taken into consideration when comparing athletes who may have been scanned at differing sites and even at the same site if the software on the machine has been updated.

#### 3.1.2. Legal and Ethical Considerations

DXA exposes athletes to low radiation doses (ranging between 0.1 and 75 µSy dependent upon manufacturer, model and scan mode used). Considering this in perspective, the effective dose of two of the most widely used commercially available DXA systems (Hologic Discovery A system and GE-Lunar iDXA), which are relatively similar in radiation exposure (4–5 µSy), is less than the average natural daily background radiation experienced (5–8 µSy per day) in the United Kingdom [67,75,77,78]. However, due to radiation exposure, there are still legal and ethical constraints surrounding the dose of radiation emitted during DXA scanning (for both athlete and operator), which despite being relatively minimal when compared to other radiographic devices, is a factor that requires consideration. For example, in the United Kingdom, scanning for research purposes requires either ethical approval at a national level (a local ethics committee is not sufficient) or a referral from a qualified medical practitioner. Moreover, whist radiation is low, there is currently considerable debate as to the maximum times per annum this technique can be utilised and it is therefore not suitable if teams wish to assess body composition regularly throughout the season, i.e., monthly. Furthermore, it would also be inappropriate to scan females who may be pregnant.

#### 3.1.3. Technical Standardisation Requirements

Despite DXA scanner hardware/software limitations and the legal/ethical considerations, whole-body DXA measurements can be a reliable measure of body composition in athletic populations when standardised protocols are used [79], with technical error of measurements of ~0.1% for total mass, ~0.4% for total LM, ~1.9% for total FM and ~0.7% for total BMC [69]. Whilst measurements should always be taken from the whole body within the limits of the bed, this can be problematic with athletes such as rugby players, who may be both wider and taller than the bed. Taller athletes cannot always fit within the ~195 cm limit of the scanning area [80], which can significantly alter the outcome results. An example of this is indicated in Table 3, where a 201 cm tall athlete was placed on the DXA scanner on the same day in a number of positions including head off/feet on, head on/feet off and head and feet on, but with knees bent to 90 degrees. These varying positions altered FM by over 3 kg and total body mass by 7 kg, with BF% ranging from 13.8% to 16.6% (Table 3). Indeed, DXA may fail to accurately assess athletes who are often considered within the ‘extremes’ of physiology, such as tall and extremely muscular athletes. Specifically, historical data have shown issues with those who have a chest depth >25 cm [81] and particularly lean athletes, where negative fat values were found for the torso [82]. Given that many sports teams often set arbitrary non-discriminatory BF% targets for athletes (often 15% with sanctions in place when this is not achieved), positioning can lead to errors and thus major consequences for the athlete. To date, there is no approved standardisation procedure for tall athletes (in our experience most teams opt for head off feet on) and we would suggest that, as a minimum, this needs to be considered and reproduced for each scan, whilst future studies should establish best practice for such situations.

Table 4 summarises a number of emerging technical considerations that must be accounted for and/or controlled to generate reliable DXA body composition data. It is important that athletes have fasted overnight when being scanned, which can be challenging when assessing large squads of individuals [79]. Given that a typical DXA scan can take 10–15 min per individual, when testing an entire sporting squad in one day it is possible that some athletes may need to be fasted until the late afternoon, which is often not feasible. Given that it is unusual for a sports team to own their own DXA unit, assessment can involve visiting a local University, which poses challenges when ensuring pre-scanning standardisation procedures. Indeed, studies have suggested that eating a meal can lead to as much as a 2.6% increase in FM [83]. Another key consideration is the effect of muscle glycogen on the reliability of DXA body composition measures. It is now common practice for athletes to periodise carbohydrate intake, commencing some training sessions with low and often competing with high muscle glycogen, utilising the ‘fuel for the work required’ concept [84,85,86,87]. Glycogen depletion significantly affects DXA results, with a 2.5% increase in LM when glycogen super compensated [83]. Conversely, this could have major implications for athletes who may feel they have lost LM, when in reality they may simply have depleted glycogen stores due to training. Similarly, creatine loading can have major effects on the reliability of the data obtained [88]. Other factors to consider include time of day, hydration status and previous exercise activity [83,89]. Careful planning is required for the use of DXA to give the best possibility of reproducible data, which from an applied perspective is often difficult to control on a regular basis. Perhaps the strongest example of the effects of diet and exercise on DXA data veracity is in a series of case studies from our research group, summarised in Table 4. These reports involved professional combat sport athletes ‘making weight’ for competition utilising acute weight loss (AWL) strategies, inclusive of the manipulation of carbohydrate intake and hydration status. Post AWL, LM and FM was reduced by 17.5% and 10.4% respectively, across a 4 day period of intense energy and water restriction. These values rebounded by 25.4% and 40.6%, respectively following a two week period of rehydration, refuelling and recovery, inclusive of total cessation of training activity [9]. In addition, when assessing another weight making athlete one day prior and following weigh in, LM and FM were both reduced by 4.0% and 5.8%, followed by a 10.0% and 4.6% rebound [10]. These values are physiologically impossible and highlight the effects that acute changes in nutrition and exercise status can have on DXA body composition data, further illustrating the importance of pre scan standardisation to enhance reproducibility of the outcome data. It is crucial coaches are aware of these limitations and create conditions where such controls can be implemented. Indeed, if it is not possible to implement such controls, it would seem unethical to subject an athlete to a radiation dose, albeit a very low concentration, if the data produced have limited validity and could result in inaccurate practical outcomes. It could therefore be argued that if it is not necessary to assess BMC and/or limb-specific measurements of FM and FFM, then skinfold thickness measures may provide a suitable and more reliable alternate in free-living conditions. Where using DXA as a technique to measure body composition, it is imperative to ensure standardised, best practice protocols are followed [69] and all information considered within the Committee on Medical Aspects of Radiation in the Environment’s (COMARE) report considered [90].

### 3.2. Skinfold Thickness Assessment

Despite the aforementioned simplicity of skinfold measurement, which makes this method popular within applied settings, there are a number of technical limitations that must be considered when employing this technique. Initially, there is an assumption of constant skin thickness and compressibility in the double fold between differing intra and inter individual measurement sites. This is also affected by the grip of the practitioner and the applied pressure of the caliper, and an athlete’s age, sex and skin temperature. However, skinfold assessment has also been shown to be the least affected method by everyday activities, ingestion of a meal and changes in hydration status [79,91]. To that end, the experience of the anthropometrist is crucial in obtaining accurate skinfold data. From our own experiences and previous reports, as little as a 1 cm difference in the assessed measurement site can have significant effects on the outcome data [92]. Even with ISAK-accredited practitioners, it is not uncommon to see large discrepancies in assessment outcomes, particularly in larger athletes, which can create issues when multiple testers perform the measurements across groups of individuals.

A key issue when utilising skinfold thickness in the applied setting is the desire for FM measurements to be reported as a BF%, which adds another layer of complexity and turns an indirect method into doubly indirect. Doubly-indirect methods incorporate regression equations by plotting results against a criterion standard to create an estimate of composition. The conundrum with these regression equations is there are currently over one hundred such formulae for the estimation of BF% from skinfold thickness measurements alone [73], and these equations have not yet been validated when tracking regular changes in body composition [93]. These formulae are also established across varying populations, using numerous protocols, with deviations in sites measured and often have intra-practitioner and criterion variability and reliability issues. This can be characterised by the use of different equations on the same set of individual data producing resounding differences (as highlighted in Table 2), where data on a Caucasian male soccer player resulted in ranges between 4 and 8% body fat dependent upon the equation used. Therefore, the conversion of skinfold thickness into a BF% should be discouraged with data presented as a sum of the 8 skinfold sites providing a more accurate and reliable outcome of body composition assessment [20,94,95]. Indeed, the sum of skinfold thicknesses has a high degree of agreement with whole-body measures from DXA [96]. However, there are some considerations with this approach. Firstly, it is not possible to further estimate FFM, which is often useful information for those in the field. The second problem is that many coaches are not familiar with being given data as a ‘sum of mm’ and often still request relativiseddata. This is compounded by limited normative data of 8 skinfold measures in athletes and thus coaches can be somewhat confused when presented with such information. We therefore present a set of normative data taken from applied practitioners working in the field, which we believe can help to address this problem (Table 5). Finally, even in ISAK-accredited practitioners, it is not uncommon to see sum of 4, 7 or 8 sites reported. This can cause confusion and make it difficult to compare data. If the field is to move away from percentage values then it is important that a widely accepted methodology is adopted, which we would suggest is the standard ISAK sum of 8 sites as presented in Table 4.

## 4. Conclusions and Recommendations for the Field

**Figure 2:**
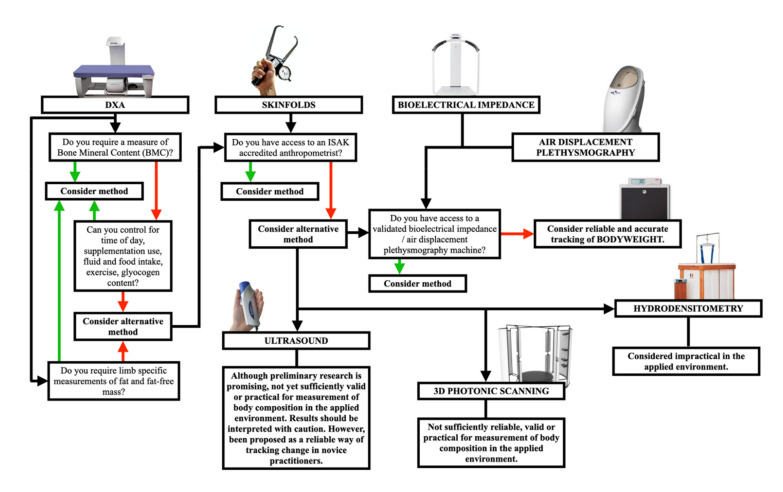
A proposed body composition method decision-making tree. Evidence base and applicability of all methods should be considered within the specific context in which they are being applied, be conducted by a suitably accredited/trained individual with all risks managed and should deliberate all points made throughout the current article prior to application of the chosen method. Green arrows indicate yes as the answer, red arrows indicate no as the answer, and black arrows indicate the potential flow of questioning when considering different methods of anthropometrical assessment.

Despite the assessment of body composition being routine practice in applied sports settings, there would appear to be nothing routine with regards to the techniques used to assess it. All of the methods discussed in this review have strengths and weaknesses, and at times may be deemed ‘best practice’ in specific athletic situations and to address specific questions. A schematic representation of the pertinent considerations that could be addressed when making a decision on the preferred method of assessing body composition can be seen in Figure 2. We would suggest where BMC needs to be examined, or when it is necessary to take limb-specific estimations of FM and FFM, then DXA appears to be the assessment tool of choice (providing the pre-scan conditions can be controlled as discussed). However, given the simplicity of the skinfold technique, the speed in which it can be implemented and assessed, the frequency of which it can be used along with the low costs associated with the method. If the goal is to simply track changes in body fatness over time, it could be argued that skinfold measures may still provide the best solution when reported as a sum of mm rather than a relative percentage value. Combined with the fact that of all of the assessments of body composition, skinfold assessments appear to be the least affected by factors that are difficult to control in athletes (food intake, hydration status, daily activity) perhaps it is now time to say in applied sports practice ‘come back skinfolds, all is forgiven’.

## Figures and Tables

**Table 1:** An overview of the different methodologies for assessing body composition in sport. The authors of the current paper met to rate each technique on a number of key consideration based upon the current balance of evidence within the scientific literature.

Method of Assessment	Evidence of Reliability	Speed of Measurement	Affordability of the Unit	Ease of Standardisation	Suitability for Sport
Hydro-Denitrometry (Two-Compartmental Doubly Indirect)	***	**	**	**	Inappropriate – lack of specialised equipment available and uncomfortable for the athlete
Air Displacement Plethysmography (Two-Compartmental Doubly Indirect)	***	***	***	***	May not be suitable to measure in-season changes to body composition and inappropriate for athletes at extremes of the BMI
Bioelectrical Impedance Spectroscopy (Multi-Compartmental Doubly Indirect)	***	*****	***	**	Useful to detect changes over time but not to measure LBM/FM and many standardisation factors to consider
Ultrasound A-Mode (Single-Compartmental (Doubly) Indirect)	**	****	****	****	Time and cost effective with good potential application in sport but needs further research
3D Photonic Scanning (Single-Compartmental (Doubly) Indirect)	Data lacking	*****	***	Data lacking	Given the lack of data in athletic populations, this method requires further study before being utilized in sport
Dual-Energy X-ray Absorptiometry (DXA) (Three-Compartmental Indirect)	*****	****	*	**	Best when segment specific LM changes, or bone density measures are required i.e. following injury or suspected low energy availability. Use heavily dependent on access and available finance with many standardisation factors to consider
Skinfold Thickness (Two-Compartmental (Doubly) Indirect)	****	****	*****	*****	Time and cost effective method to assess FM and track change over time

Classifications range between 1 * (low) and 5 ***** (high) star ratings. It should be noted that star ratings are based on ideal conditions/equipment, for example taken by an accredited, suitably trained practitioner with the best available equipment. * Low, ** Low-Medium, *** Medium, **** Medium-High, ***** High.

**Table 2:** Example of the differences observed in practice using real-world data derived from English Premier League and Women’s Super League soccer players. This includes using different DXA scanners made by the same company, on the same individuals, alongside an example of the effect of different predictive equations on collected skinfold data.

Real World DXA Data					
Participant & DXA Characteristics	Multi-ethnic backgrounds, Females, Soccer Players (*n* = 5), Age: 25 ± 5 years, Height: 167.5 ± 4.0 cm				
	SCAN 1: QDR Series Discovery A, Hologic Inc., Bedford, MA, Software Version 12.4, Weight: 64.0 ± 7.7 kg				
	SCAN 2: QDR Series Horizon A, Hologic Inc., Bedford, MA, Software Version 13.6.0.2, Weight: 64.0 ± 6.8 kg				
**Scan Details**	**Fat Mass (kg)**	**Lean Mass (kg)**	**BMC (kg)**	**Body Mass (kg)**	**Body Fat (%)**
SCAN 1	12.1 ± 2.1	50.0 ± 6.1	2.9 ± 0.2	65.0 ± 7.9	18.6 ± 1.7
SCAN 2	14.2 ± 2.0	48.0 ± 5.1	2.8 ± 0.2	63.0 ± 10.0	21.9 ± 1.6
	**Δ Fat Mass (%)**	**Δ Lean Mass (%)**	**Δ BMC (%)**	**Δ Body Mass (%)**	**Δ Body Fat (%)**
SCAN 1 vs. SCAN 2	18.0	−4.0	−2.2	−3.3	18.0
**Real World Skinfold Data**					
Participant Characteristics	ATHLETE 1: Caucasian, Male, Soccer Player, Age: 26 years, Height: 180.0 cm, Weight: 79.4 kg, Bicep, 4.0 mm; Tricep, 4.2 mm; Chest, 4.4 mm; Axilla, 5.2 mm; Subscapular, 7.6 mm; Abdominal, 7.4 mm; Supraspinale, 4.6 mm; Iliac Crest, 9.0 mm; Thigh, 4.6 mm; Calf, 4.0 mm.				
	ATHLETE 2: Caucasian, Male, Rugby Player, Age: 27 years, Height: 195.5 cm, Weight: 133.7 kg, Bicep, 6.2 mm; Tricep, 8.0 mm; Chest, 14.4 mm; Axilla, 18.4 mm; Subscapular, 25.2 mm; Abdominal, 29.8 mm; Supraspinale, 27.2 mm; Iliac Crest, 31.2 mm; Thigh, 12.5 mm; Calf, 13.4 mm.				
**Equation Used**	**Designed For**	**Sites Used**	**Calculation**	**Body Fat (%)**	
				**Athlete 1**	**Athlete 2**
Durnin & Womersley (1974) [16] & Siri (1961) [17]	Age Specific Male Population	Biceps, Triceps, Subscapular, Suoprailiac	Body Density = 1.1631 − (0.0632 × Log ΣSF)Body Fat Percentage: ((495/body density) − 450)	8.2%	23.1%
Jackson & Pollock (1978) [18] & Siri (1961) [17]	General Male Population	Chest, Abdominal, Thigh	Body Density = 1.10938 − (0.0008267 × ΣSF) + (0.0000016 × ΣSF^2^) − (0.0002574 × age)Body Fat Percentage = ((495/body density) − 450)	4.3%	16.6%
Jackson & Pollock (1978) [18]& Siri (1961) [17]	General Male Population	Chest, Axilla, Triceps, Subscapular, Abdominal, Suprailiac, Thigh	Body Density = 1.112 − (0.00043499 × ΣSF) + (0.00000055 × ΣSF^2^) − (0.00028826 × age)Body Fat Percentage = ((495/body density) − 450)	5.4%	19.4%
Withers et al., (1987) [19]& Siri (1961) [17]	Athletic Male Population	Biceps, Triceps, Subscapular, Suprailiac, Abdominal, Thigh, Calf	Body Density = 1.0988 − (0.0004 × ΣSF)Body Fat Percentage = ((495/body density) − 450)	7.3%	22.2%
Reilly et al., (2009) [20]	Athletic Male Soccer Population	Thigh, Abdominal, Triceps, Calf	Body Fat Percentage = 5.174 + (0.124 × Thigh) + (0.147 × Abdominal) + (0.196 × Triceps) + (0.130 × Calf)	8.2%	14.4%

Dual-energy X-ray absorptiometry (DXA); bone mineral content (BMC); skinfolds (SF).

**Table 3:** An example of failure to fit different athletic body types within the confinements of the DXA bed and how this affects results: (a) head on, feet off; (b) head off, feet on; (c) head on, feet on with 90° bend of the knee.

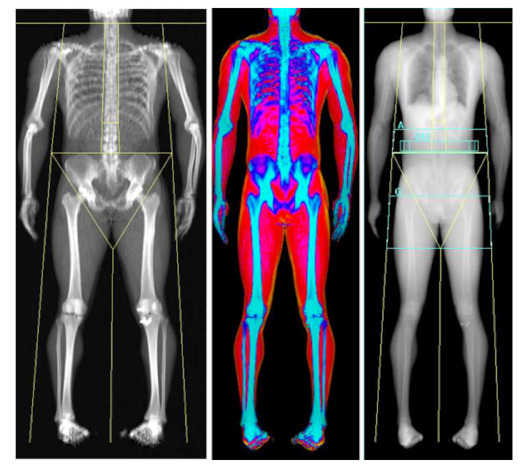 **HEAD OFF/FEET ON** **(a)**		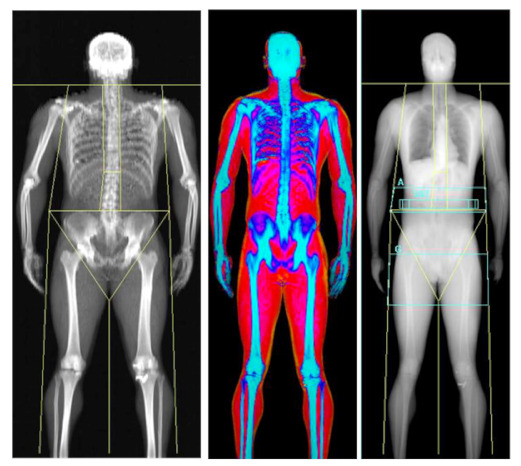 **HEAD ON/FEET OFF** **(b)**		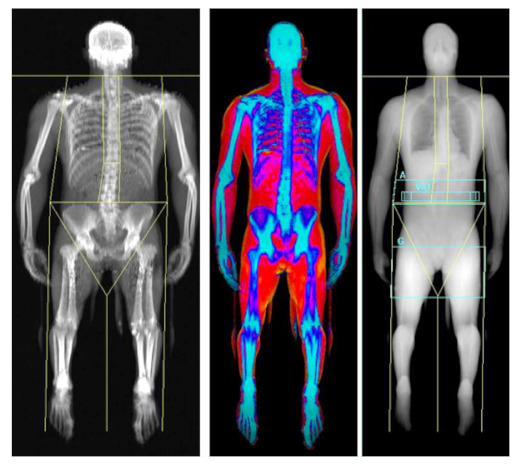 **HEAD ON/LEGS ON - 90° bend** **(c)**	
Characteristics - Caucasian, Male, Age: 35 years, Height: 201.0 cm, Weight: 103.5 kg DXA - QDR Series Horizon, Hologic Inc., Bedford, MA, USA					
**SCAN**	**Fat Mass (kg)**	**Lean Mass (kg)**	**BMC (kg)**	**Body Mass (kg)**	**Body Fat (%)**
HEAD OFF/FEET ON	13.3	79.3	4.0	96.5	13.8
HEAD ON/FEET OFF	16.6	79.4	4.1	103.0	16.6
HEAD ON/LEGS ON	14.2	79.1	4.2	97.6	14.6

All measurements collected one after the other by one of the research team. dual-energy X-ray absorptiometry (DXA); bone mineral content (BMC).

**Table 4:** A summary of differences in DXA scan results over the course of a habitual day [83]; following creatine supplementation [88]; exercise activity [89]; glycogen storage [88]; rapid weight loss and gain [9,10]; dual-energy X-ray absorptiometry (DXA); bone mineral content (BMC).

[83]	Characteristics - Race Unknown, Males, Age: 28 ± 6 years, Height: 178.0 ± 6.0 cm, Weight: 75.0 ± 9.0 kg, DXA: Lunar Prodigy, GE Healthcare, Madison, WI, USA					
[89]	Characteristics - Race Unknown, Males, Age: 30 ± 6 years, Height: 178.6 ± 6.0 cm, Weight: 80.6 ± 10.2 kg, DXA: Lunar Prodigy, GE Healthcare, Madison, WI, USA					
[88]	Characteristics - Race Unknown, Males, Age: 31 ± 6 years, Height: 182.7 ± 7.2 cm, Weight: 78.2 ± 8.8 kg, DXA: Lunar Prodigy, GE Healthcare, Madison, WI, USA					
[9]	Characteristics - Caucasian, Male, Age: 22 years, Height: 180.0 cm, Weight: 75.8 kg, DXA: QDR Series Horizon, Hologic Inc., Bedford, MA, USA					
[10]	Characteristics - Caucasian, Male, Age: 19 years, Height: 166.0 cm, Weight: 72.5 kg, DXA: QDR Series Horizon, Hologic Inc., Bedford, MA, USA					
**Effect of:**	**Δ Fat Mass (%)**	**Δ Lean Mass (%)**	**Δ BMC** **(%)**	**Δ Body Mass (%)**	**Δ Body Fat (%)**	**Reference**
**Experimental Research Derived Data**						
NO INTERVENTION (IMMEDIATE RETEST)	−0.4	0.0	0.3	0.0	0.1	[83]
HABITUAL DAY (AM TO PM, ~12h)	−1.7	0.8	0.3	0.4	0.3	[83]
HABITUAL DAY (AM TO AM, ~24h)	−0.6	−0.2	−0.2	−0.2	0.1	[83]
MEAL CONSUMPTION	2.6	1.5	0.4	1.5	−0.2	[83]
EXERCISE ACTIVITY	0.2	0.4	0.0	0.4	−0.3	[89]
CREATINE LOADING	0.4	1.1	0.0	1.3	3.3	[88]
GLYCOGEN DEPLETION	−0.2	−1.1	0.0	−1.3	−2.0	[88]
GLYCOGEN LOADING	0.5	1.8	0.0	2.3	4.5	[88]
GLYCOGEN & CREATINE LOADING	0.6	2.5	0.0	3.0	5.2	[88]
**Case Study Derived Data**						
RAPID WEIGHT DEPLETION (TIME COURSE: 4D)	−10.4	−17.5	−3.1	−12.7	3.8	[9]
RAPID WEIGHT DEPLETION (TIME COURSE: 1D)	−5.8	−4.0	−0.8	−2.8	0.0	[10]
RAPID WEIGHT Regain (TIME COURSE: 1D)	4.6	10.0	0.0	4.5	5.5	[10]
RAPID WEIGHT REGAIN (TIME COURSE: 14D)	40.6	25.4	−2.1	26.3	10.9	[9]

**Table 5:** Overview of Σ8 skinfold ranges (mm) in a variety of sports (data compiled from personal communications with peers working in elite performance). Lower, middle and upper ranges suggested are based upon typical values measured in elite sport although it must be stressed that attributing performance to skinfold measures is difficult to establish.

	Males			Females		
	Lower	Middle	Upper	Lower	Middle	Upper
Combat Athletes	35–40	40–55	55–65	45–50	50–65	65–75
Cricket Batsmen	45–55	55–65	65–70	90–100	100–120	120–140
Cricket Bowlers	40–50	50–60	60–70	75–80	80–100	100–120
Distance Running	30–40	40–45	45–55	40–55	55–70	70–85
Field Hockey	35–40	40–55	55–65	50–65	65–80	80–90
Football	40–45	45–55	55–65	60–65	65–75	75–85
Road Cycling	30–35	35–40	40–50	–	–	–
Rowing Lightweight	30–35	35–45	45–55	40–45	45–50	50–55
Rowing Openweight	35–45	45–60	60–70	55–65	65–80	80–95
Rugby Backs	40–45	45–60	60–75	55–60	60–70	70–80
Rugby Forwards	40–55	55–70	70–90	65–70	70–80	80–95
Rugby 7s	45–50	50–65	65–75	–	–	–
Swimming	40–45	45–55	55–65	55–70	70–80	80–95
Tennis	40–45	45–55	55–65	50–55	55–65	65–75

## Data Availability

No application.
